# Capnographic monitoring reduces hypoxia incidence in older patients undergoing gastrointestinal endoscopy under propofol sedation: study protocol for a multicenter randomized controlled trial

**DOI:** 10.1186/s13063-023-07208-0

**Published:** 2023-03-15

**Authors:** Qiuyue Lian, Shaoyi Chen, Xiangyang Cheng, Jie Zhang, Weifeng Yu, Renlong Zhou, Diansan Su

**Affiliations:** Department of Anesthesiology, Renji Hospital, School of Medicine, Shanghai Jiaotong University, Shanghai, China

**Keywords:** Capnographic monitoring, Hypoxia, Gastrointestinal endoscopy, Older patients, Propofol, Randomized controlled trial

## Abstract

**Background:**

Hypoxia is a very common adverse event that occurs during gastrointestinal endoscopy under sedation, especially in older patients, owing to limited reservation of heart, brain, lung, and other organs. Prolonged or severe hypoxia can cause ischemia of the coronary artery and permanent nervous system damage, and even result in death. Hence, it is imperative to reduce or prevent hypoxia during gastrointestinal endoscopy under sedation in older patients. Although several oxygen delivery methods would reduce hypoxia during this procedure, early detection of respiratory depression and early administration of intervention would be the best method to reduce or even confirm the hypoxia. Capnographic monitoring is reportedly more sensitive for detecting respiratory depression before the onset of hypoxia than the current clinical routine monitoring of pulse oxygen saturation; however, its effect is controversial. Therefore, in this study, we aimed to improve the safety of gastrointestinal endoscopy under sedation in older patients.

**Methods:**

A multicenter, randomized, single-blind, two-arm parallel-group, controlled with an active comparator, interventional superiority clinical trial will be conducted to evaluate the impact of an additional capnographic monitoring-based intervention on the incidence of hypoxia in older patients. Patients (*n* = 1800) scheduled for gastrointestinal endoscopy with propofol sedation will be randomly assigned to either a control or interventional arm, wherein standard or capnographic monitoring is implemented, respectively.

**Discussion:**

This study primarily aims to examine whether an additional capnographic monitoring-based intervention can reduce the incidence of hypoxia in older patients during gastrointestinal endoscopy under propofol and sufentanil sedation. The results of this study may extensively impact gastrointestinal endoscopy under sedation and the development of associated guidelines.

**Trial registration:**

ClinicalTrials.gov NCT05030870. Registered on September 1, 2021.

**Supplementary Information:**

The online version contains supplementary material available at 10.1186/s13063-023-07208-0.

## Administrative information

Note: the numbers in curly brackets in this protocol refer to SPIRIT checklist item numbers. The order of the items has been modified to group similar items (see http://www.equator-network.org/reporting-guidelines/spirit-2013-statement-defining-standard-protocol-items-for-clinical-trials/).

**Table:** 

Title {1}	Capnographic monitoring reduces the incidence of hypoxia in older patients undergoing gastrointestinal endoscopy under propofol sedation: Study protocol for a multicenter randomized controlled trial
Trial registration {2a and 2b}.	ClinicalTrials.gov , NCT05030870. Registered on September 1, 2021.
Protocol version {3}	The protocol version is 2.2, which was approved in November 22, 2021.
Funding {4}	This study was supported by the National Nature Science Foundation of China (Nos. U21A20357) .
Author details {5a}	Authors Qiuyue Lian, Shaoyi Chen, Xiangyang Cheng, Jie Zhang, Weifeng Yu, Renlong Zhou, Diansan Su Department of Anesthesiology, Renji Hospital, School of Medicine, Shanghai Jiaotong University, Shanghai, China Email address: Qiuyue Lian: l18724464815@163.com Shaoyi Chen: docteurchensy@163.com Xiangyang Cheng: 1647384344@qq.com Jie Zhang: q150981@qq.com Weifeng Yu: ywf808@yeah.net Renlong Zhou: renlongzhou@shsmu.edu.cn Diansan Su: diansansu@yahoo.com Corresponding author Diansan Su Department of Anesthesiology, Renji Hospital, Shanghai Jiaotong University School of Medicine, 160 Pujian Road, Shanghai, 200127, China Email address: Diansan Su: +862168383702, Email:diansansu@yahoo.com Co-corresponding author Renlong Zhou Department of Anesthesiology, Renji Hospital, Shanghai Jiaotong University School of Medicine, 160 Pujian Road, Shanghai, 200127, China Email address: Renlong Zhou: +862168383702, Email:renlongzhou@shsmu.edu.cn
Name and contact information for the trial sponsor {5b}	Sponsor:The National Nature Science Foundation of China (Nos. U21A20357) Grant recipient: Diansan Su Address:Department of Anesthesiology, Renji Hospital, Shanghai Jiaotong University School of Medicine, 160 Pujian Road, Shanghai, 200127, China Tel:+862168383702
Role of sponsor {5c}	The funder had no role in the analyses and interpretation of the results or writing of the manuscript.

## Introduction

### Background and rationale{6a}

The sedation rate of gastrointestinal endoscopy varies in different countries. In China, the overall sedation rate for gastrointestinal endoscopy is ~50% [1], whereas in the USA, >98% gastrointestinal endoscopy procedures are performed under sedation [2, 3]. Cardiopulmonary complications, especially hypoxia, are the most common complications that occur during gastrointestinal endoscopy under sedation. Prolonged or severe hypoxia can cause ischemia of the coronary artery and permanent nervous system damage or even result in death [4, 5]. Thus, determining a method to reduce hypoxia incidence in gastrointestinal endoscopy under sedation is crucial.

Compared with the current clinical routine monitoring of pulse oxygen saturation (SpO_2_), capnographic monitoring can detect hypoventilation and respiratory depression earlier, thereby helping in providing earlier intervention [6]. Therefore, it is believed that capnographic monitoring would reduce the incidence of hypoxia. Nevertheless, whether capnographic monitoring should be routinely performed during gastrointestinal endoscopy under sedation with propofol remains controversial.

There are two major studies regarding this subject; however, their results are discrepant. The first investigation, a randomized, controlled study (ColoCap Study) conducted by Beltz et al., confirmed that additional capnographic monitoring of ventilatory activity in ASA I–III patients reduces the incidence of hypoxia during propofol sedation for colonscopy [7]. However, the second investigation, a cohort study, reported that additional capnographic monitoring did not improve patient safety or satisfaction, but it did increase the cost [8]. The fundamental problem was that the initial oxygen flow in the ColoCap Study was only 2 L/min, which is inconsistent with that in clinical practice (3–4 L/min). This inconsistency resulted in a hypoxia incidence of >50% in the control group. Another limitation of the ColoCap Study was that the depth of sedation between the two groups may have been inconsistent. Although the cohort study was designed to closely mimic clinical practice, it did not yield positive results. These conflicting results indicate that there is insufficient evidence regarding routine capnographic monitoring in patients.

Older patients comprise a vulnerable group who are more susceptible to hypoxia during gastrointestinal endoscopy performed under drug-induced sedation [9, 10]. However, to the best of our knowledge, whether this vulnerable group requires routine monitoring for capnography has not been investigated. Therefore, this study was designed to improve the security of gastrointestinal endoscopy under sedation in older patients.

### Objectives {7}

Our aim is to investigate whether an additional capnographic monitoring-based intervention can reduce hypoxia incidence in older patients undergoing gastrointestinal endoscopy under sedation. Our primary objective is to measure the incidence of hypoxia (75% ≤ SpO_2_ < 90% for < 60 s). Our secondary objective is to measure the incidences of subclinical respiratory depression (90% ≤ SpO_2_ < 95%), severe hypoxia (SpO_2_ < 75% or 75% ≤ SpO_2_ < 90% for ≥60 s), and other adverse events (AEs) recorded via tools proposed by the World Society of Intravenous Anesthesia International Sedation Task Force. We predict that additional capnographic monitoring of ventilatory activity will reduce hypoxia incidence during propofol sedation for gastrointestinal endoscopy in older patients. Our aim is to provide credible evidence regarding reduced hypoxia incidence in older patients.

### Trial design {8}

This study is a multicenter, randomized, single-blind, two-arm parallel-group, controlled with an active comparator, interventional superiority clinical trial. We intend to evaluate the impact of an additional capnographic monitoring-based intervention on hypoxia incidence in older patients.

This trial was conducted under the recommendations of the Standard Protocol Items: Recommendations for Interventional Trials (SPIRIT). The trial was registered on ClinicalTrials.gov (No. NCT05030870) on September 1, 2021. We presented the trial registration data in the form of Supplemental data.

## Methods: participants, interventions, and outcomes

### Study setting {9}

This study will enroll ~1800 participants from the Renji Hospital Shanghai Jiao Tong University School of Medicine, Henan Provincial People's Hospital, and Qilu Hospital of Shandong University. The ethics committee of the Renji Hospital Shanghai Jiao Tong University School of Medicine approved and supported this clinical trial (KY-2021014).

### Eligibility criteria {10}

This clinical trial has three centers that recruited patients. A summary of the inclusion and exclusion criteria is presented in Table 1.

**Table 1:** Inclusion/exclusion criteria

Inclusion criteria	Exclusion criteria
1) Aged ≥65 and <80 years	1) Coagulation disorders or a tendency of nose bleeding
2) Scheduled to undergo gastrointestinal endoscopy procedure with sedation	2) An episode/exacerbation of congestive heart failure that requires a change in medication, diet, or hospitalization from any cause in the past 6 months
3) Signed the informed consent form	3) Severe aortic stenosis or mitral stenosis
4) American Society of Anesthesiologists (ASA) classification I-II	4) Cardiac surgery involving thoracotomy (e.g., coronary artery bypass graft, valve replacement surgery) in the past 6 months
	5) Acute myocardial infarction in the past 6 months
	6) Acute arrhythmia (including any tachycardia or bradycardia) with fluid of hemodynamics instability
	7) Diagnosed with chronic obstructive pulmonary disease or current other acute or chronic lung disease requiring supplemental chronic or intermittent oxygen therapy
	8) Preexisting bradycardia (heart rate <50/min), or hypoxia (SaO_2_ < 90%)
	9) Need supplemental oxygen because of preexisting diseases
	10) Emergency procedure or surgery
	11) Multiple trauma
	12) Upper respiratory tract infection
	13) Allergy to propofol or tape and adhesives

### Who will take informed consent? {26a}

Trained anesthesiologists will explain this trial to the potential participants in detail, and the informed consent form will be provided. Participants can decide whether they wish to participate in the trial after sufficient time to deliberate. Subsequently, the participant or his/her trustee or guardian can sign the informed consent form, and they can withdraw at any time during the trial. Following this, baseline data will be collected from the patients, and they will be randomly allocated using the central random system. Participants can contact our team if they have any health concerns during the trial. The entire process of recruiting participants and obtaining their consent by the members of the research team will be performed in accordance with good clinical practice (GCP). In case of any AEs during the trial related to the study procedure or not, the researchers will immediately report to the director in charge of the clinical trial of the research institution and contact Professor Diansan Su.

### Additional consent provisions for collection and use of participant data and biological specimens {26b}

Not applicable as no participant data and biological specimens were collected or used in ancillary studies.

## Interventions

### Explanation for the choice of comparators {6b}

For the choice of comparators, the central randomization system will be used for each study site. Following randomization, the participants will receive either standard monitoring (Arm2) or additional capnography (Arm1). The comparator in this trial is standard monitoring alone (Arm2).

### Intervention description {11a}

Capnography-blinded arm (Arm2) and capnography-open arm (Arm1) constitute the interventions for the participants in this trial. The capnography-blinded arm involves standard monitoring, and the capnography-open arm involves additional capnography.

In both the groups, standard monitoring will include determining the heart rate, SpO_2_, electrocardiogram, and noninvasive blood pressure of the selected patients. In the capnography-open arm, a sampling line will be connected to a bedside portable monitor (Capnostream 20; Medtronic, Inc.) to ensure that the capnographic data of the patients are available for the additional noninvasive assessment of ventilation. A nasal cannula equipped with an oral sampling port to accommodate mouth breathers provided 2 L/min oxygen and continuously sampled CO_2_ content of both inspired and expired patient gas. The sampling line was connected to a portable bedside monitor (Capnostream 20; Medtronic, Inc.) displaying the time-based CO_2_ graphic waveform, numerical CO_2_ partial pressure (mmHg), derived respiratory rate, and SpO_2_ via integrated pulse oximetry. The height, shape, and rhythm of the capnogram provide a real-time assessment of ventilatory function. In the presence of supplemental oxygen during inspiration, the samples essentially contain no CO_2_, and the samples represent alveolar CO_2_ concentration during expiration, with a small amount of gas containing no CO_2_ from the patient physiologic dead space. For alveolar hyperventilation, the samples comprise reduced to no CO_2_. In the capnography-blinded arm, no sampling line will be connected to the bedside portable monitor, and the capnographic data of the patients will not be visible to ensure that only the integrated pulse oximetric readout of the monitor is visible.

Table 2 lists the AEs of anesthesia and sedation.

**Table 2:** Adverse events of anesthesia and sedation

**Step 1:Was there one or more adverse events associated with this sedation encounter?**				
□ No, this form is now complete.	□ Yes, fill out the remainder of the form below.			
**Step 2: Please DESCRIBE the adverse event(s). Check all that apply.**				
**Minimal risk descriptors**	**Minor risk descriptors**		**Sentinel risk descriptors**	
□ Vomiting/retching	□ Oxygen desaturation (75–90%) for < 60s		□ Oxygen desaturation, severe (<75% at any time) or prolonged (<90% for >60s)	Other, specify below
□ Sub-clinical respiratory depression^a^	□ Apnoea not prolonged		□ Apnoea, prolonged (>60s)	
□ Muscle rigidity, Myoclonus	□ Airway obstruction		□ Cardiovascular collapse/shock^g^	
□ Hypersalivation	□ Failed sedation^e^		□ Cardiac arrest/absent pulse	
□ Paradoxical response^b^	□ Allergic reaction without anaphylaxis			
□ Recovery agitation^c^	□ Bradycardiat^f^			
□ Prolonged recovery^d^	□ Tachycardia^f^			
	□ Hypotension^f^			
	□ Hypertension^f^			
	□ Seizure			
**Step 3: Please note the INTERVENTIONS performed to treat the adverse events(s). Check all that apply.**				
**Minimal risk**	**Minor risk**	**Moderate risk**	**Sentinel intervention**	
□ No intervention performed	□ Airway repositioning	□ Bag valve mask-assisted ventilation	□ Chest compressions	Other, specify below
□ Tactile stimulation	□ Tactile stimulation	□ Laryngeal mask airway	□ Tracheal intubation	
□ Additional sedative(s)	Or the administration of:	□ Ora/nasal airway	Or the administration of:	
□ Antiemetic	□ Supplemental oxygen, new or increased	□ CPAP	□ Neuromuscular block	
□ Antihistamine	□ Antisialogogue	Or the administration of:	□ Pressor/epinephrine	
		□ Reversal agents	□ Atropine to treat bradycardia	
		□ Rapid i.v.fluids		
		□ Anticonvulsant i.v.		
**Step 4: Please note the OUTCOME of the adverse events(s). Check all that apply.**				
**Minimal risk outcome**	**Moderate risk outcome**		**Sentinel outcome**	
□ No adverse outcome	□ Unplanned hospitalization or escalation of care^h^		□ Death	Other, specify below
			□ Permanent neurological deficit	
			□ Pulmonary aspiration syndrome^i^	
**Step 5: Assign a SEVERITY rating to the adverse event(s) associated with this sedation encounter.**				
If there are any options checked in the Sentinel columns above, then this is a Sentinel adverse event^j^.				
If the most serious option(s) checked above are Moderate risk, then this is a Moderate risk adverse event^k^.				
If the most serious option(s) checked above are Minor risk, then this is a Minor risk adverse event^l^.				
If the most serious option(s) checked above are Minimal risk, then this is a Minimal risk adverse event^m^.				

Footnotes:

^a^ “Sub-clinical respiratory depression” is defined as capnographic abnormalities suggesting respiratory depression that do not manifest clinically

^b^ “Paradoxical response” is defined as unanticipated restlessness or agitation in response to sedatives

^c^ “Recovery agitation” is defined as abnormal patient affect or behaviors during the recovery phase that can include crying, agitation, delirium, dysphoria, hallucinations, or nightmares

^d^ “Prolonged recovery” is defined as failure to return to baseline clinical status within 2 hours

^e^ “Failed sedation” is defined as inability to attain suitable conditions to humanely perform the procedure

^f^ Alteration in vitals signs (bradycardia, tachycardia, hypotension, hypertension) is defined as a change of >25% from baseline

^g^ “Cardiovascular collapse/shock” is defined as clinical evidence of inadequate perfusion

^h^ Examples of “escalation of care” include transfer from ward to intensive care, and prolonged hospitalization

^i^ “Pulmonary aspiration syndrome” is defined as known or suspected inhalation of foreign material such as gastric contents into the respiratory tract associated with new or worsening respiratory signs

^j^ “Sentinel” adverse events are those critical enough to represent real or serious imminent risk of serious and major patient injury. Once recognized, they warrant immediate and aggressive rescue interventions. Once clinically concluded, they warrant immediate reporting within sedation care systems and the highest level of peer scrutiny for continuous quality improvement

^k^ “Moderate” adverse events are those that, while not sentinel, are serious enough to quickly endanger the patient if not promptly managed. Once clinically concluded, they warrant timely reporting within sedation care systems and periodic peer scrutiny for continuous quality improvement

^l^ “Minor” adverse events are those encountered periodically in most sedation settings, and that pose little threat given appropriate sedationist skills and monitoring

^m^ “Minimal” adverse events are those that alone present no danger of permanent harm to the patient

### Criteria for discontinuing or modifying allocated interventions {11b}

If participants request to withdraw from the trial, we will discontinue the allocated intervention for a given trial participant.

### Strategies to improve adherence to interventions {11c}

Adherence to interventions primarily refers to patient self-management adherence.

### Relevant concomitant care permitted or prohibited during the trial {11d}

No concomitant care or interventions will be permitted during this trial.

### Provisions for posttrial care {30}

If a participant suffers harm from this trial, he/she will receive financial compensation accordingly. The amount of compensation will be determined jointly by consulting the relevant departments and the participant.

### Outcomes {12}

#### Primary outcome measures

The primary outcome of this study is the incidence of hypoxia (75% ≤ SpO_2_ < 90% for <60 s) in the two groups from the beginning to the end of the operation.

#### Secondary outcome measures

The secondary outcomes comprise the following:

1. The incidence of subclinical respiratory depression (90% ≤ SpO_2_ < 95%) in the two groups from the beginning to the end of the operation.
2. The incidence of severe hypoxia (SpO_2_ < 75% or 75% ≤ SpO_2_ < 90% for ≥60 s) in the two groups from the beginning to the end of the operation.
3. The incidence of other AEs recorded via tools proposed by the World Society of Intravenous Anesthesia International Sedation Task Force.

### Participant timeline {13}

Table 3 presents the schedule for the enrollment, interventions, assessments, and visit of the participants.

**Table 3:** Schedule of the major study events

Project	Gastroscopy diagnosis and treatment period(Visit 1)					
	Arrive the examination room	sedative induction	Procedure start	Procedure over	participants wakes up	Leave the examination room
**Baseline data**						
Informed consent	**×**					
Medical history	**×**					
Inclusion / exclusion criteria	**×**					
Demographic data	**×**					
Vital signs	**×**	**×**	**×**	**×**	**×**	**×**
Physical examination	**×**					
**Research outcome measures**						
Hypoxia		**×**	**×**	**×**		
Sub-clinical hypoxia		**×**	**×**	**×**		
Severe hypoxia		**×**	**×**	**×**		
Adverse event		**×**	**×**	**×**		
**Research drug**						
Study randomization	**×**					
Calculate drug dosage						**×**
**Others**						
Gastroscope procedure time				**×**		
Combined medication	**×**	**×**	**×**	**×**	**×**	**×**

When the participants will enter the gastrointestinal endoscopic operating room, they will be screened for eligibility by the investigator. If they meet the inclusion criteria but not the exclusion criteria, the investigator will provide them with the fully informed consent form. After signing the informed consent form, the patients will be allocated to either the capnographic monitoring group or the control group via the central randomization system.

In the capnographic monitoring group, the criteria for apnea is the absence of exhaled CO_2_; altered ventilation is defined as end-tidal CO_2_ reduced by more than half of the baseline, as shown by the capnogram; and the definition of hypoxia is 75%<SpO_2_ < 90%, <60s. In the control group, hypoxia is defined as SpO_2_ <90%.

In both the groups, any sign of apnea, altered ventilation, or hypoxia that prompts an intervention will comprise (i) increasing oxygen supplementation, (ii) a chin lift or jaw thrust maneuver, (iii) insertion of the oropharyngeal or nasopharyngeal airway with a chin lift or jaw thrust maneuver, (iv) artificial mask ventilation, and (v) tracheal intubation.

The duration of surgery is defined as the time from the beginning to the end of the gastrointestinal endoscopy, excluding the time of resuscitation.

### Sample size {14}

Our previous study revealed that hypoxia incidence in patients during gastrointestinal endoscopy with propofol sedation was ~8 % [11]. The anticipated effect size of additional capnographic monitoring was 50%, implying that the hypoxia incidence power analysis assumes a reduction from 8% to 4%. The results of a conventional analysis were compared between the capnography-open and capnography-blinded groups to detect differences in proportions (hypoxia). We use PASS 11.0, randomization 1:1, power of 1 − β = 0.90, and a two-sided α level of 5%. We assume a 10% dropout rate. Thus, the results revealed that ~1800 patients would be required.

### Recruitment {15}

The schedule of the major study events for each study visit is presented in Table 3. This study included older patients who are scheduled to undergo gastrointestinal endoscopy with propofol sedation. The patients (65 ≤ age <80 years) who met the inclusion criteria were preliminarily screened by the investigators and recruited by distributing recruitment materials to patients and their families. Additionally, we put up recruitment posters in the endoscopy centers explaining the advantages of our trial. The nasal catheters that will be used in our trial are free, and the trial is beneficial for participants. We estimate that the trial will easily recruit enough participants according to the number of patients admitted to the endoscopy centers per day.

The entire process of recruiting participants and obtaining their consent by the members of the research team will be consistent with GCP.

## Assignment of interventions: allocation

### Sequence generation {16a}

In this trial, we used stratified blocked randomization to design the central randomization system. Risk factors for hypoxia during sedated gastrointestinal endoscopy include patient factors, sedation factors, and endoscopic operation factors. Patient factors mainly include preexisting cardiopulmonary disease, obesity, and advanced age. The incidence of hypoxia during sedated gastrointestinal endoscopy varies among patients with different underlying conditions. However, the subjects in our trial were all ASA I–II patients and did not include patients with ASA III-IV, which means that the trial did not involve patients with previous severe cardiopulmonary disease. Additionally, the number of obese patients in the older population in China is extremely low, and stratification by obesity would provide little benefit. Our study only included ASA I–II older patients aged 65 to 80 years, who we can assume have similar physical conditions, and the incidence of hypoxia during sedated gastrointestinal endoscopy is approximately the same. Therefore, we did not stratify them by age, and only central stratification was used in our trial. Complete baseline data will be collected from participants, including name, gender, date of birth, etc. After assessing patient eligibility for inclusion, his/her informed consent will be obtained. We will randomly assign the participants in a ratio of 1:1 to the standard or additional capnographic monitoring group according to the allocation sequence of the central randomization system. The length of a random sequence is not fixed, and 4, 6, and 8 are random.

### Concealment mechanism {16b}

After obtaining the signed informed consent, the participants will be randomly assigned to the standard or additional capnographic monitoring group according to the central randomization system. The random results, random number, and their relationship with groups will be kept confidential from the participants throughout the trial. The same nasal cannula with a CO_2_-collecting device will be used in both groups and connected to the capnographic monitoring device. The participants will not be aware of their own and others’ grouping before, during, and following gastrointestinal endoscopy.

### Implementation {16c}

Designated doctors will generate the allocation sequence, enroll participants, and assign participants to interventions.

## Assignment of interventions: blinding

### Who will be blinded {17a}

After the interventions are assigned, only the trial participants will be blinded. The results in the central randomized system will be kept confidential from the participants throughout the entire trial. The same nasal cannula will be used in both the standard and additional capnographic monitoring group, and the sampling line will be connected to a portable beside monitor. The researcher will ensure that participants are not aware of their own or other’s assignment.

### Procedure for unblinding if needed {17b}

As the trial is single-blind, patients interested in knowing their group could be informed by the investigator following the analysis of results.

## Data collection and management

### Plans for assessment and collection of outcomes {18a}

This study is an internal multicenter clinical trial. All data collection physicians will be specially trained by assessors. We will conduct regular online meetings to share the progress of the trial and discuss the problems encountered during the project. Moreover, we will conduct field visits to subcenters for quality control. We shall also regularly organize the trial data to check for any missing data, and to promote data quality, we intend to apply other methods and call the participants as well.

### Plans to promote participant retention and complete follow-up {18b}

Not applicable, as the trial will not involve follow-up; thus, we have no plans to promote participant retention and complete the follow-up.

### Data management {19}

The case report form (CRF) of the respective patients will be entered and/or filled in for all the collected patient data during this clinical trial. The study number, subject number, date of subject information, and informed consent will be appropriately documented in the patient CRF. We will archive the source data as per GCP guidelines. The data manager will be responsible for data processing and will conduct regular monitoring according to the sponsor’s standard operating procedures to ensure that the dates are adequate, accurate, and complete. The source data lock will occur only after the completion of the quality assurance procedures.

### Confidentiality {27}

Participant information will be confidential and managed according to the Data Protection Act, NHS Caldecott Principles, The Research Governance Framework for Health and Social Care, and the conditions of Research Ethics Committee approval. The confidentiality of the data collected during the course of the research will be strictly maintained, and only the members of the trial team (or individuals from the sponsor organization or center sites relevant to the trial) will be allowed to access the data. All documents containing patient information are stored in a specific cabinet in the anesthesia department, locked and keyed for safekeeping. The permission of the principal investigator is required for the removal or access of the data. The participants will be allocated an individual trial identification number and their details will be stored in a secure database. This database is maintained by professional researchers, and only the principal investigator has access to the data set. The anonymized trial data are not to be shared with other researchers.

### Plans for collection, laboratory evaluation, and storage of biological specimens for genetic or molecular analysis in this trial/future use {33}

Not applicable as no biological specimens were collected as part of this trial.

## Statistical methods

### Statistical methods for primary and secondary outcomes {20a}

#### Data selection for statistical analysis

1. Full analysis set (FAS): According to the principle of intention-to-treat analysis, the full analysis set will include all subjects who are randomized to the study and receive the study treatment.
2. Per-protocol set (PPS): The PPS population will include all FAS patients without major protocol deviations that influence the evaluation of primary outcomes, such as the different dosages of sufentanil during the induction or lack of primary outcome data. The efficacy analysis will be performed on the FAS and PPS.
3. Safety analysis set (SAS): The safety population will comprise all subjects who receive the study treatment. Analyses of safety data in the study will be based on the safety population.

#### Statistical analysis plan

All statistical analyses in this trial will be programmed and calculated using Statistical Package for the Social Sciences version 23.0 (IBM Inc., Armonk, NY, USA). The normally distributed baseline continuous data will be represented by mean (standard deviation [SD]) and compared by using an unpaired *t*-test. The non-normally distributed continuous data will be presented as median (interquartile range [IQR]) and compared by using Mann–Whitney *U* test. We will use χ^2^ test, continuity correction *χ*^2^ test, or Fisher’s exact test to analyze the categorical variables. We intend to compare the incidences of hypoxia, subclinical hypoxia, severe hypoxia, and total hypoxia of the participants during gastrointestinal endoscopy under sedation via *χ*^2^ test, continuity correction *χ*^2^ test, or Fisher’s exact test.

#### Additional analyses

Safety analysis: general safety evaluations will be based on the incidence and type of AEs. Safety variables will be tabulated and presented for all the patients in safety sets. AEs will be coded using the tools proposed by the World Society of Intravenous Anesthesia International Sedation Task Force. The number (%) of subjects with any AEs will be summarized and compared via *χ*^2^ test, continuity correction *χ*^2^ test, or Fisher’s exact test.

### Interim analyzes {21b}

Not applicable as we have no plans to conduct any interim analyses, and no one has the rights to access these interim results and decide to terminate the trial.

### Methods for additional analyses (e.g., subgroup analyses) {20b}

The association between the baseline characteristics and intervention and the risk of total hypoxia cumulative incidence was examined using univariable- and multivariable-adjusted logistic regression models. The results were presented as the odds ratios and corresponding 95% confidence intervals.

### Methods in the analysis to handle protocol nonadherence and any statistical methods to handle missing data {20c}

Statistical analysis was performed based on intention-to-treat. Regardless of protocol adherence, the results of the outcome analyses will be randomly analyzed. The frequency and type of missingness of all the variables will be screened. If missingness is >5% of any variable, we will use multiple imputations. Complete case analysis will be performed as a sensitivity analysis, in case of missing data and imputation.

### Plans to provide access to the full protocol, participant-level data, and statistical code {31c}

Not applicable as we have no plans to provide access to the full protocol, participant-level data, and statistical code.

## Oversight and monitoring

### Composition of the coordinating center and trial steering committee {5d}

Diansan Su, Jiangqiang Zhang, and Jianbo Wu are the members of the trial steering committee, and Diansan Su is responsible for preparing and revising the protocol and disseminating any changes. Renlong Zhou and Weifeng Yu are responsible for overseeing the study design and protocol and interpreting the study findings. Qiuyue Lian and Shaoyi Chen are responsible for coordinating data collection and analysis and writing the scientific manuscript. Xiangyang Cheng and Jie Zhang are responsible for overseeing any statistical analyses and ensuring that the study implementation on the floor follows the protocol.

### Composition of the data monitoring committee, its role, and reporting structure {21a}

We do not have composition of the data monitoring committee (DMC).

### Adverse event reporting and harms {22}

The nasal cannula with a port that is used for collecting exhaled CO_2_ samples, which are consequently used for capnographic monitoring, is similar to the original nasal cannula and does not have any additional risks. To date, to the best of our knowledge, there has been no evidence that this study may cause any risk or discomfort to the participants.

We will record any AEs that occur during the clinical trial, regardless of whether these events were associated with the intervention. Additionally, all the expected and unexpected trial-related AEs will be reported in the trial publications.

### Frequency and plans for auditing trial conduct {23}

The investigators shall maintain all study data according to GCP requirements. The original study data and information will be retained for at least 5 years following trial completion. Data security and monitoring reports will be submitted to the ethical committee every 3 months.

### Plans for communicating important protocol amendments to relevant parties (e.g., trial participants, ethical committees) {25}

This clinical trial will be conducted according to the ethical committee approval. Any problem or protocol modifications during the trial will be communicated to the ethical committees, trial participants, trial registries, journals, and regulators in a timely manner. The ethical committee’s consent will be required to change the protocol.

### Dissemination plans {31a}

The participants, healthcare professionals, the public, and other relevant groups in the form of articles will communicate the results of this trial.

## Discussion

This study is the first randomized controlled trial designed to confirm the utility of capnographic monitoring in older patients during gastrointestinal endoscopy under sedation. The proportion of older patients who undergo gastrointestinal endoscopy under sedation is increasing annually worldwide. Ensuring the safety of older patients during gastrointestinal endoscopy under sedation is thus crucial.

Hypoxia is the most common and severe complication that occurs during gastrointestinal endoscopy under sedation, with the primary cause of hypoxia being hypoventilation due to propofol administration. Older patients are more susceptible to respiratory depression and hypoxia than adult patients [12, 13].

The early detection of respiratory depression in patients and timely and effective intervention measures can reduce the occurrence of severe hypoxia, hypercapnia, and even cardiac arrest, thereby improving patient prognosis [14]. Capnography monitoring is more sensitive and real-time than SpO2 in reflecting respiratory depression before the onset of hypoxia [15–17].

Currently, no unified conclusion has been drawn on whether end-tidal CO2 monitoring can effectively reduce the occurrence of hypoxia during endoscopy [18–23]. There is insufficient evidence for routine capnographic monitoring in all patients during sedated gastrointestinal endoscopy, and there have been few recent studies to add to the scant literature on this topic. Our study design avoids some of the limitations of the other studies and demonstrates more closeness to clinical settings. Our study is expected to provide a higher level of evidence and improve the safety of gastrointestinal endoscopy under sedation in older patients.

Therefore, future research should seek to resolve some of the limitations of this study. First, this was a single-blinded clinical study, which might cause potential bias. Second, our trial is not international, and the participating institutions of our experiment are all Grade III Class A general hospitals in China. The medications or methods of sedation vary among different institutions and countries. Fortunately, the selected intravenous anesthetics are widely used owing to their clinical compatibility, and the data obtained in our study could be represented in most clinical settings. Third, only older patients with ASA grades I–II were included in our trial, and those with ASA grades III–IV were excluded. We planned another group to be explored in the next study for older patients with higher ASA classifications. Therefore, further capnography monitoring research in gastrointestinal endoscopy under sedation is warranted.

## Trial status

Trial registration: ClinicalTrials.gov, NCT05030870. Registered on September 1, 2021. The protocol version is 2.2, which was approved in November 22, 2021. This study was started on September 1, 2021, and the recruitment phase will last until December 2023.

## Supplementary Information

**Additional file 1.** SPIRIT checklist.

## Data Availability

Because of Chinese data protection rules and regulations, the participant-level data set cannot be made publicly available. If requested, the statistical code is available.

## Abbreviations

SpO_2_: Hemoglobin oxygen saturation
CRF: Case report form
GCP: Good clinical practice
REC: Research Ethics Committee
FAS: Full analysis set
PPS: Per-protocol set
SAS: Safety analysis set
AEs: Adverse events
ITT: Intention-to-treat
DMC: Data monitoring committee
CO_2_: Carbon dioxide
RCT: Randomized controlled study
